# Genetic characterization of an H5N6 avian influenza virus with multiple origins from a chicken in southern China, October 2019

**DOI:** 10.1186/s12917-021-02903-z

**Published:** 2021-05-28

**Authors:** Feng Wen, Jing Yang, Jinyue Guo, Congying Wang, Qing Cheng, Zheng Tang, Kaijian Luo, Sheng Yuan, Shujian Huang, Yong Li

**Affiliations:** 1 College of Life Science and Engineering, Foshan University, Foshan, 528231 Guangdong China; 2 College of Animal Science and Technology, Jiangxi Agricultural University, Nanchang, 330045 Jiangxi China; 3 College of Veterinary Medicine, South China Agricultural University, Guangzhou, 510642 Guangdong China

**Keywords:** Avian influenza viruses, H5N6, HPAIV, East Asian-Australasian flyway

## Abstract

**Background:**

Highly pathogenic avian influenza viruses (HPAIVs) of H5 subtype pose a great threat to the poultry industry and human health. In recent years, H5N6 subtype has rapidly replaced H5N1 as the most predominate HPAIV subtype circulating in domestic poultry in China. In this study, we describe the genetic and phylogenetic characteristics of a prevalent H5N6 strain in Guangdong, China.

**Results:**

Nucleotide sequencing identified a H5N6 subtype HPAIV, designated as A/chicken/Dongguan/1101/2019 (DG/19), with a multibasic cleavage site in the hemagglutinin (HA). Phylogenetic analysis revealed DG/19 was a reassortant of H5N1, H5N2, H5N8, and H6N6 subtypes of avian influenza viruses. A number of mammalian adaptive markers such as D36N in the HA were identified.

**Conclusions:**

Our results showed that HPAIV H5N6 strains still emerge in well-managed groups of chicken farms. Considering the increasing prevalence of H5N6 HPAIV, and the fact that H5N6 HPAIVs are well adapted to migratory birds, an enhanced surveillance for the East Asian-Australasian flyway should be undertaken to prevent potential threats to the poultry industry and human health.

**Supplementary Information:**

The online version contains supplementary material available at 10.1186/s12917-021-02903-z.

## Background

Avian influenza viruses (AIVs) are a group of enveloped viruses characterized with an eight-segmented, single-stranded, negative-sense RNA genome [1]. AIVs can be categorized into subtypes based on the antigenicity of two surface glycoproteins, hemagglutinin (HA) and neuraminidase (NA). To date, 16 HA subtypes and 9 NA subtypes of AIVs are reported [2]. In addition, according to the severity of disease in chickens caused by AIVs, AIVs are classified into highly pathogenic avian influenza viruses (HPAIVs) and low pathogenic avian influenza viruses (LPAIVs) [3]. HPAIVs, including subtypes of H5 and H7, which code for a furin-sensitive multibasic cleavage site (−RRKKR-) in the HA [4, 5], are capable of inducing systemic infections in multiple tissues and causing dramatic economic losses in the poultry industry.

In 1996, the first HPAIVs of H5N1 subtype (A/goose/Guangdong/1/1996 (H5N1)) was isolated from an outbreak affecting domestic geese in Foshan city, Guangdong province, China. A year later, the first human clinical respiratory case of H5N1 was reported in Hong Kong, China [6, 7]. Since then, the H5N1 virus has spread across to spread to Southeast Asia, the Middle East, Africa, and Europe, undergoing a number of genetic reassortments [8–11]. In 2014, the first H5N6 strain was isolated from backyard poultry flocks in Guangdong province, China [12]. Subsequently, HPAIV H5N6 became one of the major subtypes of the H5 clade 2.3.4.4. To date, since the first case of human infection with HPAIV H5N6 in Sichuan province in April 2014 [13], more than 23 cases of infection and 9 deaths were recorded (www.who.int/influenza/human_animal_interface). During the past several years, the HPAIV H5N6 subtype has continuously evolved and has replaced H5N1 as one of the main HPAIV subtypes in domestic poultry in China [13–16]. A more recent study suggested that the positive rate of H5N6 in live poultry markets (LPM) is 7.87%, which became the second most dominant subtype currently circulating in LPMs in China [16]. Moreover, human infection of HPAIV H5N6 subtype even had a higher mortality than H5N1 subtype based on the clinical statistical data thus far [17]. Therefore, the HPAIV H5N6 subtype contributes as a major threat to domestic poultry and human health.

In this study, we report the genetic and phylogenetic characteristics of a HPAIV H5N6 strain isolated (October 2019) from chicken farms in Dongguan, Guangdong province, China.

## Results

Primers targeting the conserved region of M segment were used to detect the influenza A virus in the liver and spleen of chickens (Supplementary Figure 1) and a band of 226 bp was observed (Supplementary Figure 2A). All 8 viral segments (PB2, 2280 bp; PB1, 2280 bp; PA, 2152 bp; HA, 1701 bp; NP, 1497 bp; NA, 1380 bp; M, 982 bp; NS bp, and 823 bp) (Supplementary Figure 2B) were successfully amplified and sequenced by cloning to the pMD 18-T vector. The results revealed that the AIV in this study belongs to a H5N6 subtype, which was designated as A/chicken/Dongguan/1101/2019 (H5N6)(DG/19). DG/19 contained six basic amino acid residues at HA cleavage site (RERRRKR↓GLF) (Table 1), which suggested a high pathogenicity phenotype in chickens.

**Table 1:** Molecular characteristics of H5 isolates associated with pathogenicity

Virus	Subtype	Clade	Pathotype	HA			NA			PB2		PB1
				Cleavage site	RBS		stalk deletion	274	294	23	627	473
					186	226–228						
A/Anhui/1/2005	H5N1	2.3.4	HPAI	RERRRKRGLF	N	QSG	YES	H	N	T	E	V
A/Hunan/1/2009	H5N1	2.3.4.1	HPAI	RERRRKRGLF	N	QSG	YES	H	N	T	E	V
A/goose/Yunnan/6193/2006	H5N1	2.3.4.2	HPAI	RERRRKRGLF	N	QSG	YES	H	N	T	E	V
A/avian/Hong_Kong/2372/2007	H5N1	2.3.4.3	HPAI	RERRRKRGLF	N	QSG	YES	H	N	T	E	V
A/duck/Hunan/2.06_YYGK78J3-OC/2018	H5N6	2.3.4.4	HPAI	RERRRKRGLF	N	QRG	NO	H	N	T	E	V
A/chicken/Dongguan/1101/2019	H5N6	2.3.4.4	HPAI	RERRRKRGLF	N	QRG	YES	H	N	T	E	V
A/muscovy duck/Japan/AQ-HE30-77C2/ 2018	H5N6	2.3.4.4	HPAI	RERRRKRGLF	N	QRG	YES	H	N	T	E	V

**Fig. 1:**
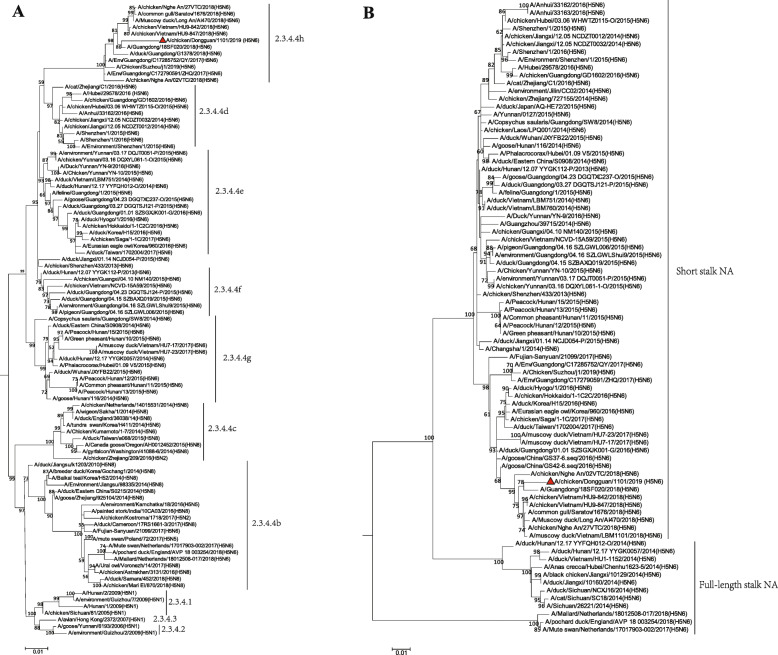
The maximum likelihood phylogenetic trees for the hemagglutinin (HA) and neuraminidase (NA) genes of the A/chicken/Dongguan/1101/2019 (H5N6)(DG/19) in this study. Phylogeny analysis was performed using the maximum likelihood method with 1000 bootstrap replicates in MEGA 7. The DG/19 in this study is indicated with red triangles. **a** Phylogenetic tree of HA gene for DG/19 suggested it belongs to clade 2.3.4.4. **b** Phylogenetic tree of NA gene for DG/19 suggested it belongs to Eurasian lineage. The isolates described in this study was marked with red triangle

The nucleotide sequences of DG/19 were aligned with the reference strains deposited in Global Initiative on Sharing Avian Influenza Data (GISAID, www.gisaid.org) and GeneBank by ClustalW. Subsequently, the phylogenetic trees for HA (Fig. 1a) and NA (Fig. 1b) genes were constructed by the maximum likelihood method. Our phylogenetic results showed that the DG/19 HA belong to clade 2.3.4.4. A further comparison with the reference sequence of the sub-clades of 2.3.4.4 suggested DG/19 belongs to the 2.3.4.4 h subclade. The internal genes of DG/19 had high identity with strains isolated from ducks in 2017–2018 in Hunan by a recent study [16]. The HA gene has the highest nucleic acid homology with A/duck/Hunan/1.12_YYGK 72H3-OC/2018(H5N6) (98.35%). Furthermore, it is worth noting that the NA gene of DG/19 has high nucleic acid homology with A/muscovy duck/Japan/AQ-HE30-77C2/ 2018 (H5N6) (97.89%). The sequence alignment suggested DG/19 had Q142H, N89S, D36N, D193N mutations in the HA compared to the closest reference strain A/duck/Hunan/1.12_YYGK72H3-OC/2018 (H5N6).

**Fig. 2:**
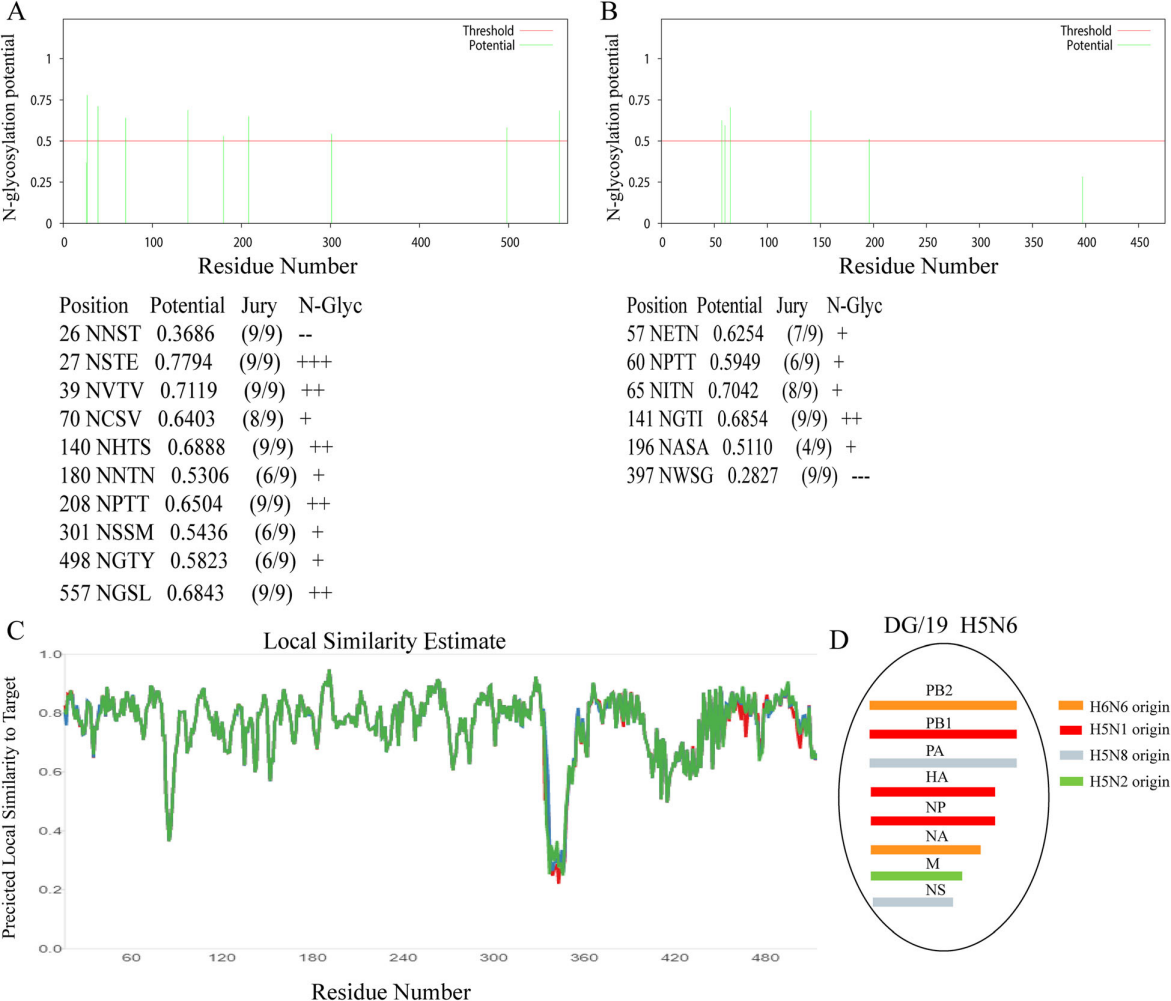
N-linked Glycosylation analysis and structural similarity prediction. The potentials of N-linked glycosylation site of HA (**a**) and NA (**b**) of the A/chicken/Dongguan/1101/2019 (H5N6)(DG/19) were calculated with NetNGlyc 1.0 Server (http://www.cbs.dtu.dk/services/NetNGlyc/) based on the consensus N-X-S/T motif. **c** The local similarity to the template 6PCX with the highest sequence similarity was predicted with by the SWISS-MODEL server. **d** The origins of the eight gene segments (PB2, PB1, PA, HA, NP, NA, M, and NS) of DG/19. Different colors represent different virus origins

We further analyzed the glycosylation sites by NetNGlyc 1.0 server, the data revealed that the DG/19 has eight potential glycosylation sites at positions 27, 39, 70, 140, 180, 301, 497, and 556 within the HA protein (Fig. 2a). The glycosylation site at the 158 site has been lost due to the T160A substitution. Meanwhile, a total of five potential glycosylation sites at positions 57, 60, 65, 141, and 196 within the NA protein were identified (Fig. 2b). In addition, the 3D structure model of HA of DG/19 was predicted by the SWISS-MODEL server and the predicted local similarity to the target with the highest sequence similarity (6PCX) was shown in Fig. 2c.

Further analysis of internal genes revealed that the PB2 gene segment had high identity with those recent H5N6 isolates in China, which was likely originated from H6N6 AIVs as the NA did. The PA and NS gene segments suggested a H5N8 origin. In addition, the M gene segment of DG/19 showed the highest similarity to the M gene of H5N2 AIVs. The phylogenetic trees for internal genes were shown in Supplementary Figure 3. These results suggest DG/19 is a reassortant of H5N1, H5N2, H5N8, and H6N6 AIVs (Fig. 2d), which might happen through the East Asian-Australasian flyway [18].

## Discussion

Southern China has been considered as a hot spot for the generation of novel AIVs. In this study, we reported the genetic characterization of a recent H5N6 HPAIV strain isolated from chickens in southern China. In addition, novel mutations such as D36N, which is one of the six common amino acid substitutions of H2 subtype AIV from mallards to swine [19], are emerging from H5N6 HPAIVs. These observations indicate a potential mammalian adaptation of H5N6 AIV. Fortunately, our results showed that DG/19 possessed 226Q and 228G (H3 numbering) in the HA, which suggested a typical avian receptor preference and were consistent with previous studies [16]. However, DG/19 has 222Q and 227R in the HA, which has been shown to facilitate virus binding to fucosylated sialosides [20]. Sialyl Lewis^X^ glycans (Neu5Gcα2-3Galβ1–4(Fucα1–3)GlcNAcβ) were shown on the respiratory track of terrestrial birds but not on the intestinal tract of ducks [21, 22]. The 222Q and 227R on HA of DG/19 suggested that H5N6 AIV has been well adapted to terrestrial birds, which made H5N6 AIV easier to adapt to mammalian hosts. The 226Q and loss of the glycosylation site at 158 suggested DG/19 prefers avian-type receptors [13, 16].

In addition, a number of novel mutations in the HA of H5N6 HPAIV were identified, including Q142H, N89S, and D193N. It has been suggested that the D193N mutation of H10N7 virus promoted virus binding to α2,6-linked sialic acid receptors without an impair of binding to α2,3-linked sialic acid receptors [23]. However, the biological effects of those mutations need further investigation.

The NA protein of DG/19 has a 11-aa stalk deletion at residues 59–69, which has been shown to enhance virulence in mammals [24]. Moreover, the L473V substitution was observed in PB1 of DG/19, which was associated with the improved replication of AIV in mammalian cells [25].

## Conclusion

In this study, we report the genetic characteristics of a recent HPAI H5N6 (DG/19) strain from chickens in southern China. Our results revealed HPAIV H5N6 strain circulating in south China is potentially a reassortment between H5N1, H5N2, H5N8, and H6N6 AIVs, highlighting the importance of continuous surveillance of AIVs through the East Asian-Australasian flyway. Further studies are needed to investigate the biological role of those novel mutations identified in this study.

## Methods

### Surveillance of avian influenza virus in domestic poultry

The routine surveillance of AIV was performed in domestic poultry in Guangdong, China. In October 2019, an outbreak of AIV was reported in a chicken farm in Dongguan, Guangdong province. The liver and spleen samples collected from chickens were homogenized 3 cycles by Precellys Evolution Super Homogenizer (France) at 6000 rpm for 15 s. The viral RNA extraction and reverse transcription was performed as previously described [18, 26]. Briefly, viral RNA was extracted by Body Fluid Viral DNA/RNA Miniprep kit (Axygen, China) according to the manufacturer’s instructions. The Maxima H Minus First Strand cDNA Synthesis Kit (Thermo Fisher Scientific, USA) with a Uni12 primer (5’-AGCGAAAGCAGG-3’) was used for reverse transcription. The present of AIV was confirmed by RT-PCR with a universal primer (M7F: 5′-CTTCTAACCGAGGTCGAAACG-3′, M232R: 5′-GTCTACGCTGCAGTCCTCGCT-3′) targeting the conserved region of M segment.

### Virus sequencing

All 8 gene segments of DG/19 were successfully amplified by Phusion Hot Start II High-Fidelity PCR Master Mix (Thermo Fisher Scientific, USA) with a set of universal primers described by Hoffmann et al. [27]. The temperature cycle parameters were 98 °C for 30 s, followed by 35 cycles of 98 °C for 10 s, 53 °C for 30 s, and 72 °C for 2 min, with a final extension at 72 °C for 10 min. The PCR products were purified by using Gene JET Extraction Kit (Thermo Fisher Scientific, USA) according to the manufacturer’s instructions. Subsequently, the purified PCR segments were incubated for 15 min at 72 °C with Premix Taq (Takara, Japan), then subcloned into the pMD-18 T vector and sequenced by Sanger sequencing.

### Phylogenetic analysis

A total of 95 representative H5N6 strains from Global Initiative on Sharing Avian Influenza Data (GISAID, www.gisaid.org) or Influenza research database (IRD, www.fludb.org) were selected for molecular evolutionary analyses. The full length of HA (1701 bp) and NA (1380 bp) genes were aligned by ClustalW and phylogenetically clustered by maximum likelihood method with 1000 bootstrap replicates in MEGA7.

### Structural and N-linked glycosylation site prediction

The 3D structure model of HA of DG/19 was analyzed by the SWISS-MODEL server (https://swissmodel.expasy.org/) [28]. In brief, the HA sequence of DG/19 was blasted through the SWISS-MODEL library and the template 6PCX with the highest sequence similarity was selected for the model building. In addition, the potential of N-linked glycosylation sites based on the consensus N-X-S/T motif was calculated as previously described [26].

## Supplementary Information

**Additional file 1: Figure S1.** Clinical pictures of chickens in this study.

**Additional file 2: Figure S2.** The amplification of conserved M gene segments (A) and full genome of A/chicken/Dongguan/1101/2019 (DG/19).

**Additional file 3: Figure S3.** The maximum likelihood phylogenetic trees for the PB2(a), PB1(b), PA(c), NP(d), M(e), and NS(f) gene segments of A/chicken/Dongguan/1101/2019 (DG/19). The virus detected in this study was indicated in red star. The scale bars represent the number of substitutions per nucleotide.

## Data Availability

The viral sequences obtained in this study are deposited in GenBank under the accession numbers: MW314745, MW314746, MW314795, MW314796, MW314797, MW314798, MW314799, and MW320522.

## Abbreviations

AIVs: Avian influenza viruses
HA: Hemagglutinin
NA: Neuraminidase
HPAIVs: Highly pathogenic avian influenza viruses
LPAIVs: Low pathogenic avian influenza viruses
GISAID: Global Initiative on Sharing Avian Influenza Data
DG/19: A/chicken/Dongguan/1101/2019 (H5N6)
LPMs: Live poultry markets
