# Supplementary figures and images for: Genetic characterization of an H5N6 avian influenza virus with multiple origins from a chicken in southern China, October 2019
Source: BMC Vet Res. 2021 May 28;17:200. doi: 10.1186/s12917-021-02903-z (PMC8161609; doi:10.1186/s12917-021-02903-z)

## Slide 1
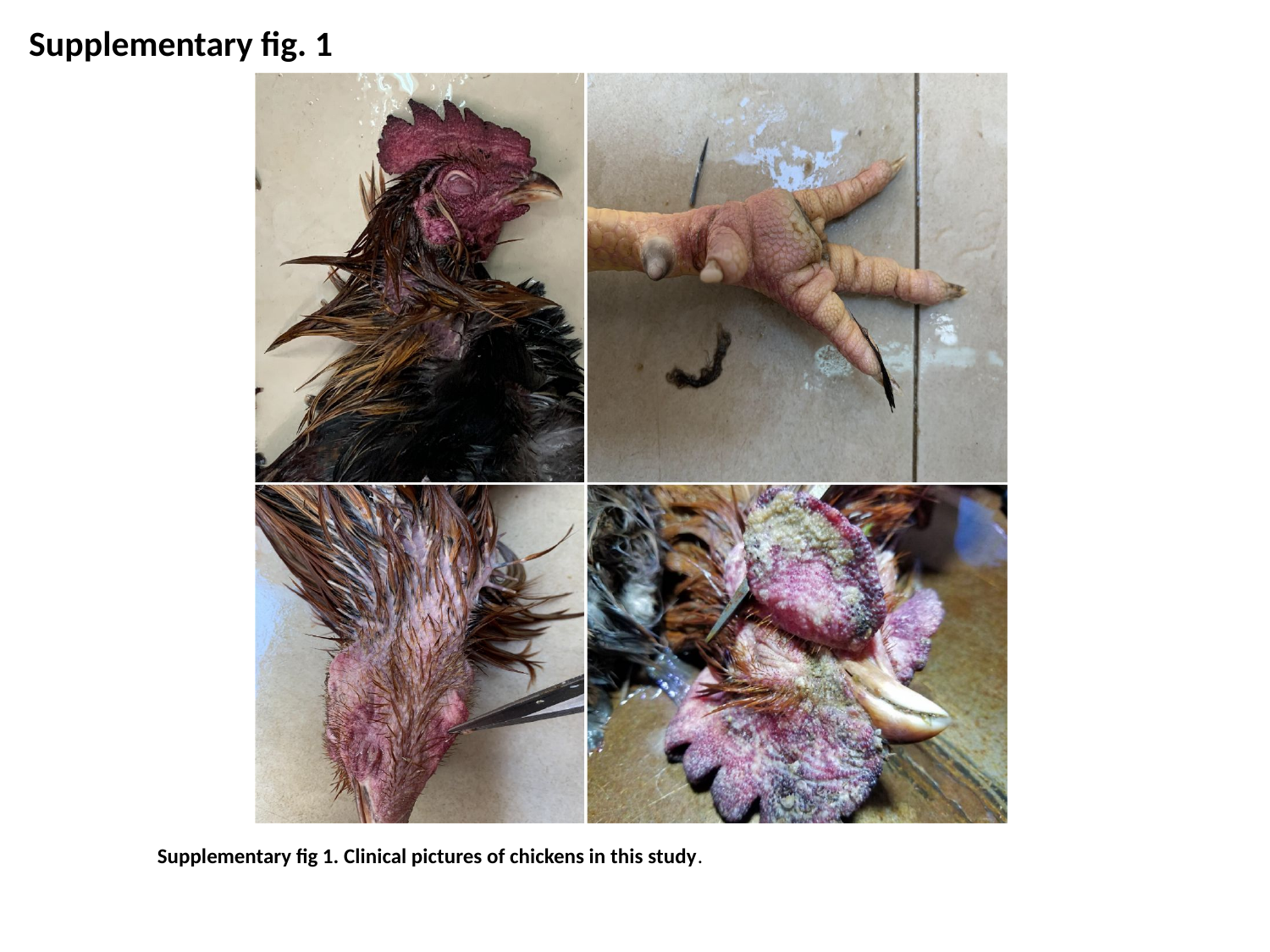

Supplementary fig. 1
Supplementary fig 1. Clinical pictures of chickens in this study.

Supplement: Supplementary file 1 — Additional file 1: Figure S1. Clinical pictures of chickens in this study. [file 12917_2021_2903_MOESM1_ESM.pptx]
