# Supplementary material for: Genetic characterization of an H5N6 avian influenza virus with multiple origins from a chicken in southern China, October 2019
Source: BMC Vet Res. 2021 May 28;17:200. doi: 10.1186/s12917-021-02903-z (PMC8161609; doi:10.1186/s12917-021-02903-z)
Supplement: Supplementary file 2 — Additional file 2: Figure S2. The amplification of conserved M gene segments (A) and full genome of A/chicken/Dongguan/1101/2019 (DG/19). [file 12917_2021_2903_MOESM2_ESM.pptx]

## Slide 1
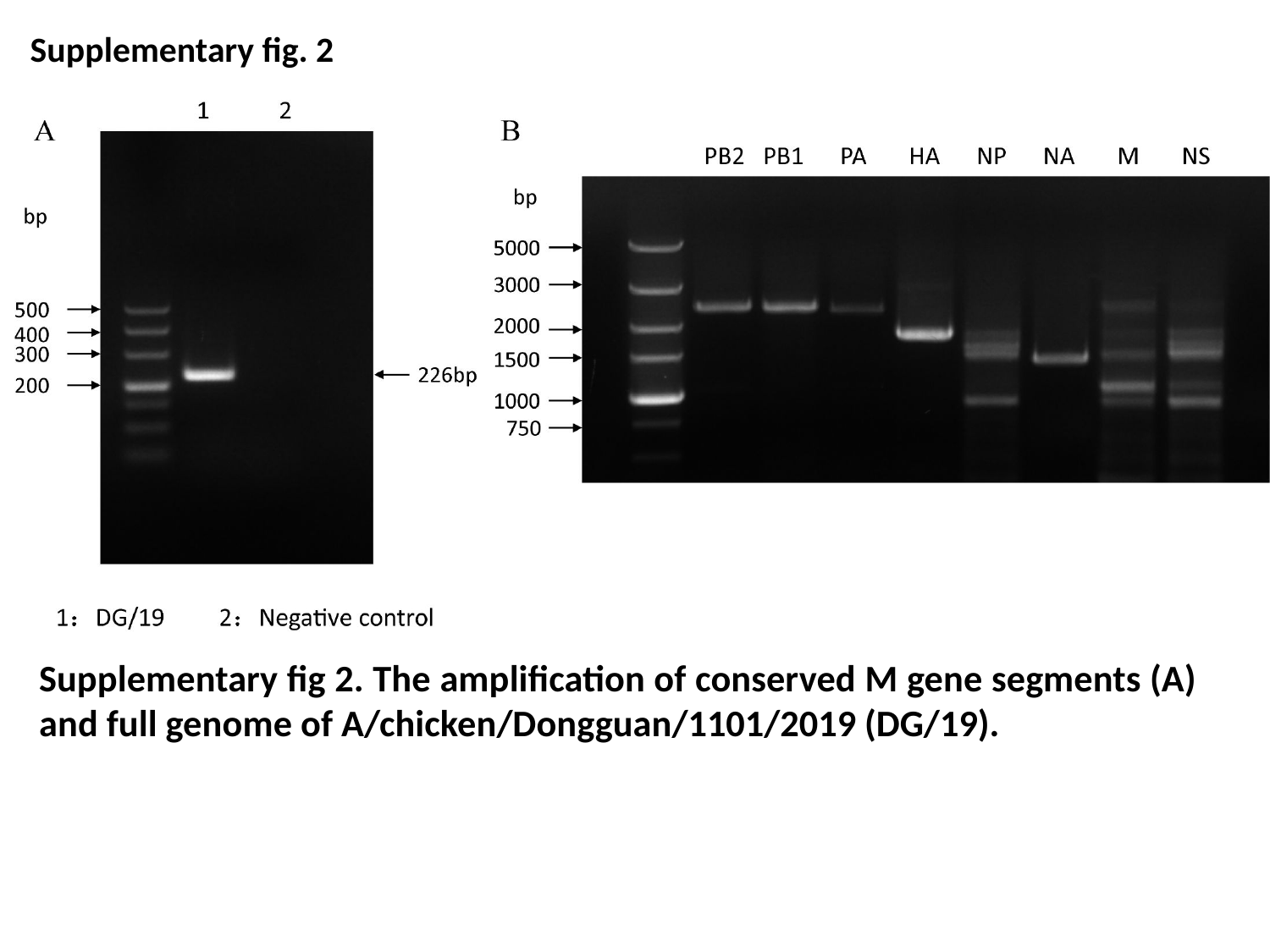

Supplementary fig. 1
(a) PB2
Supplementary fig. 2
Supplementary fig 2. The amplification of conserved M gene segments (A) and full genome of A/chicken/Dongguan/1101/2019 (DG/19).
