# Supplementary material for: Genetic characterization of an H5N6 avian influenza virus with multiple origins from a chicken in southern China, October 2019
Source: BMC Vet Res. 2021 May 28;17:200. doi: 10.1186/s12917-021-02903-z (PMC8161609; doi:10.1186/s12917-021-02903-z)
Supplement: Supplementary file 3 — Additional file 3: Figure S3. The maximum likelihood phylogenetic trees for the PB2(a), PB1(b), PA(c), NP(d), M(e), and NS(f) gene segments of A/chicken/Dongguan/1101/2019 (DG/19). The virus detected in this study was indicated in red star. The scale bars represent the number of substitutions per nucleotide. [file 12917_2021_2903_MOESM3_ESM.pptx]

## Slide 1
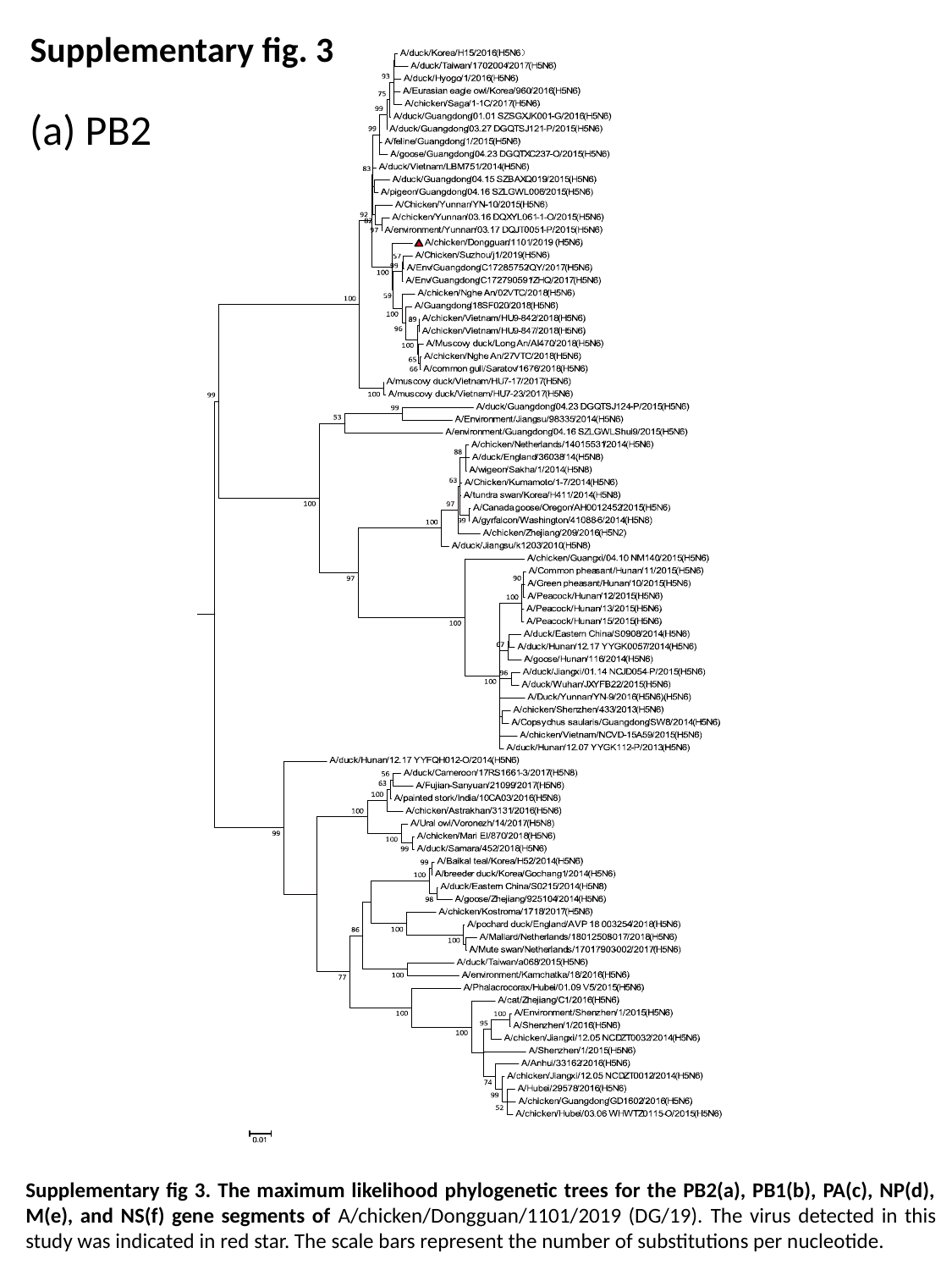

Supplementary fig. 3
(a) PB2
Supplementary fig 3. The maximum likelihood phylogenetic trees for the PB2(a), PB1(b), PA(c), NP(d), M(e), and NS(f) gene segments of A/chicken/Dongguan/1101/2019 (DG/19). The virus detected in this study was indicated in red star. The scale bars represent the number of substitutions per nucleotide.
Supplementary fig 1. The maximum likelihood phylogenetic trees for the PB2(a), PB1(b), PA(c), NP(d), M(e), and NS(f) gene segments of A/chicken/Dongguan/1101/2019 (DG/19). The virus detected in this study was indicated in red star. The scale bars represent the number of substitutions per nucleotide.

## Slide 2
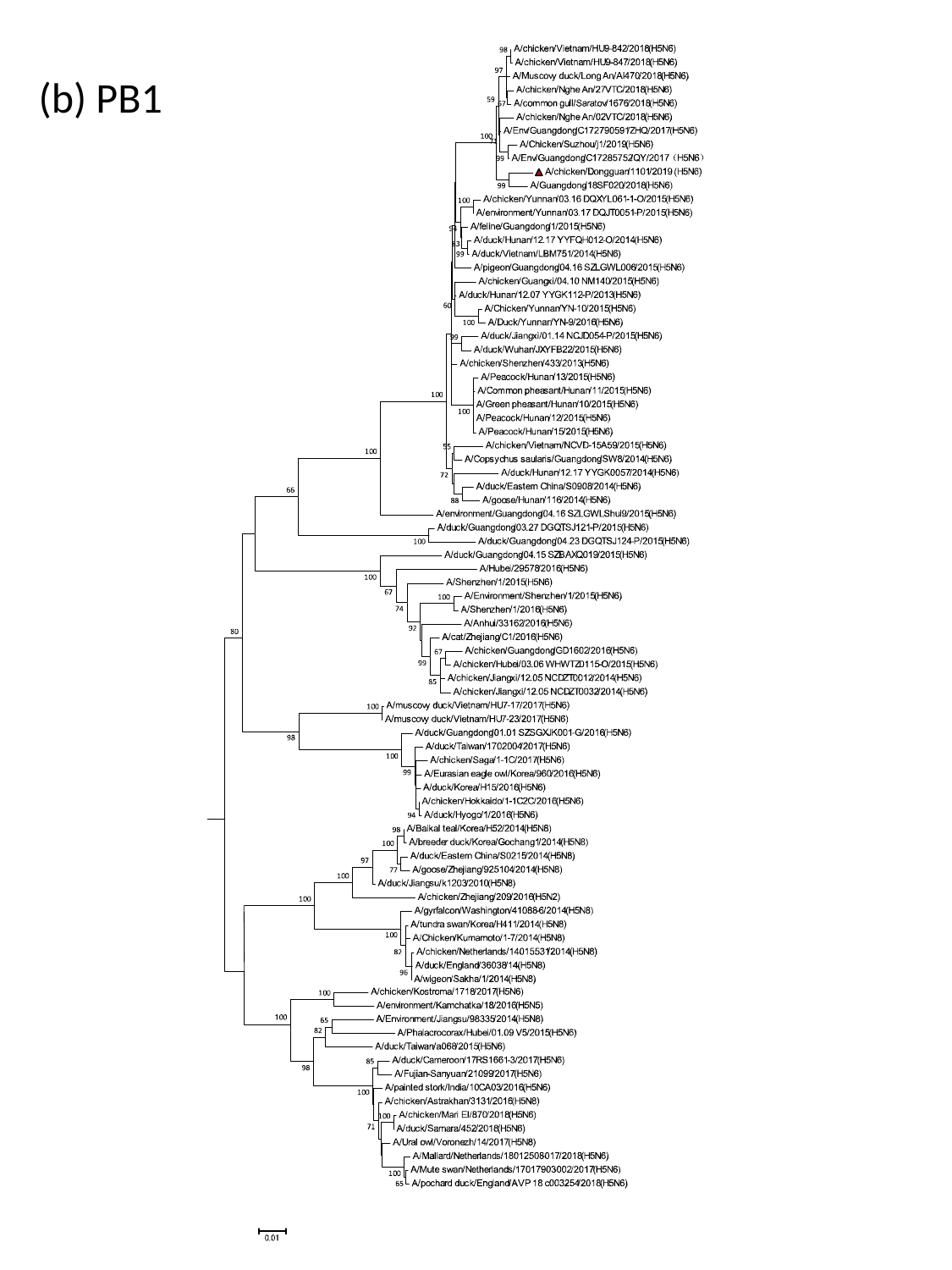

(b) PB1

## Slide 3
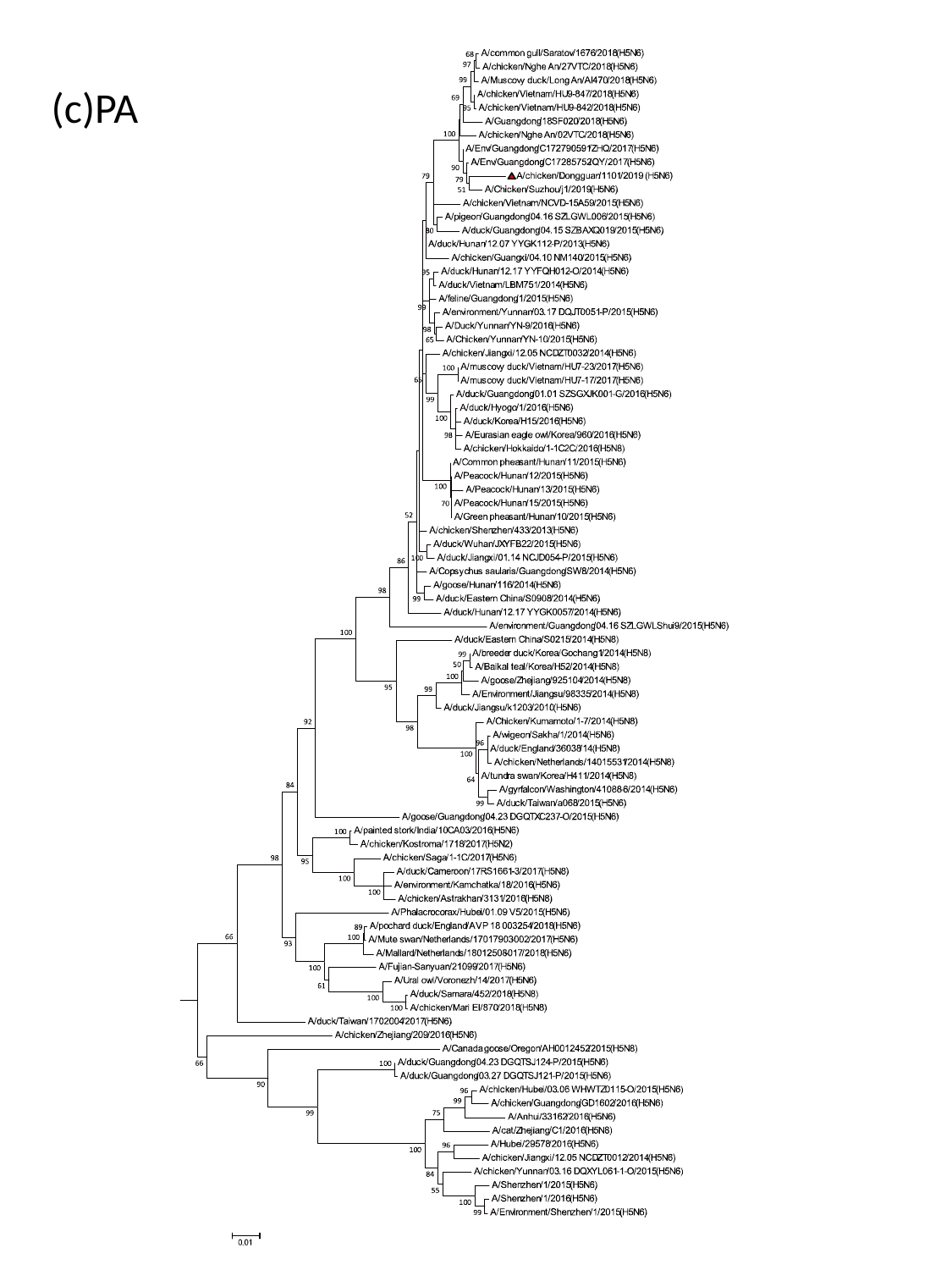

(c)PA

## Slide 4
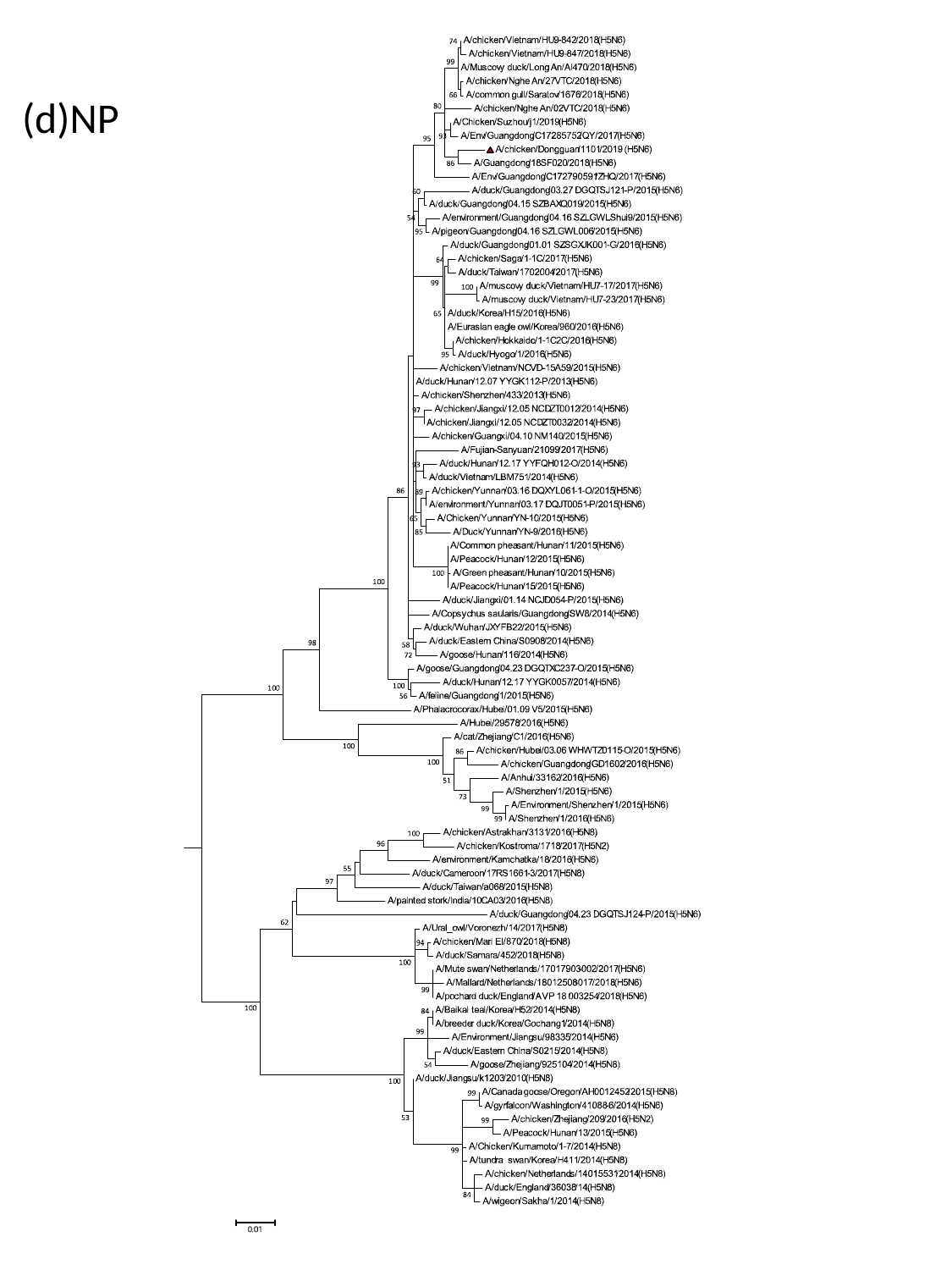

(d)NP

## Slide 5
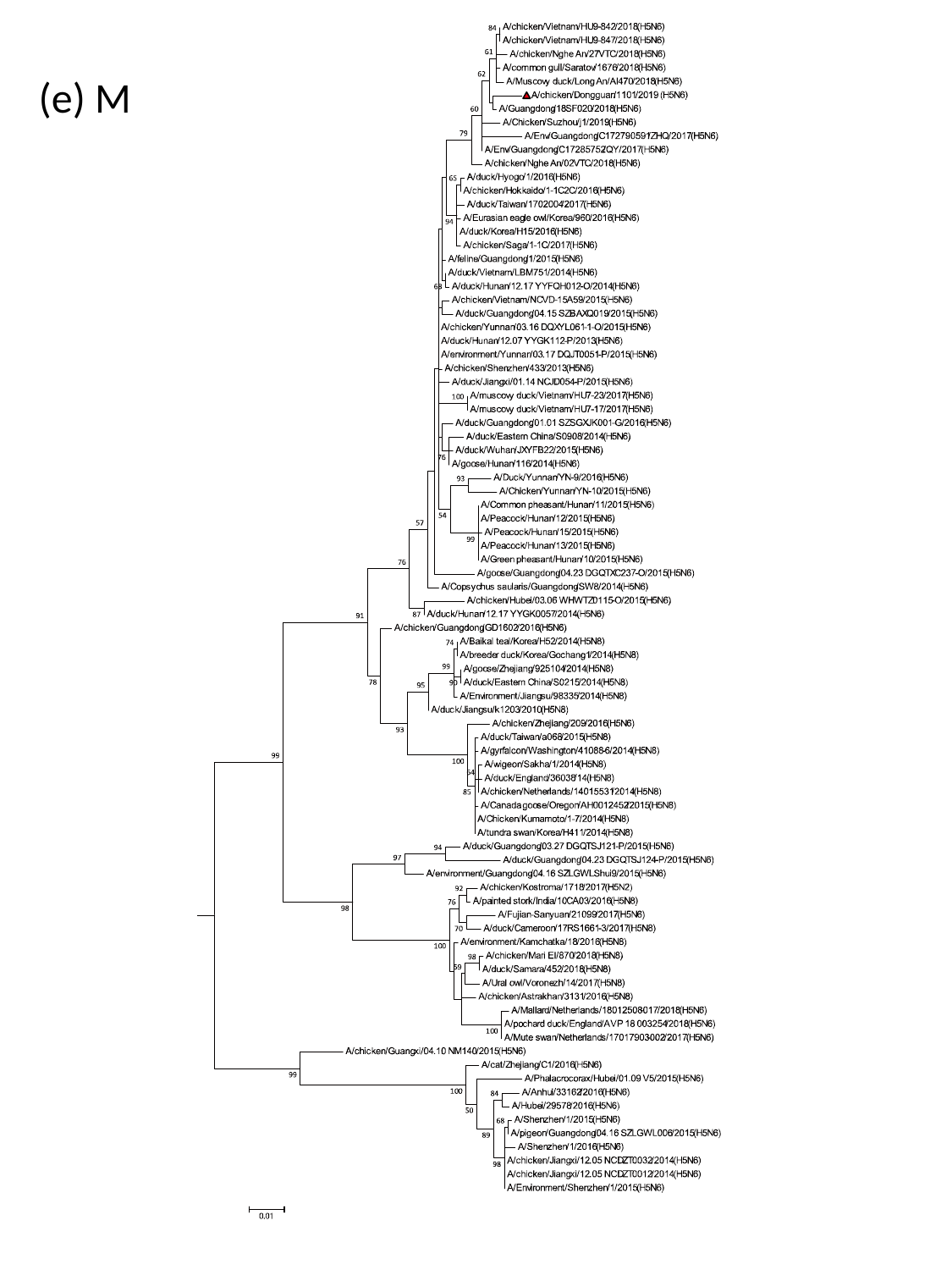

(e) M

## Slide 6
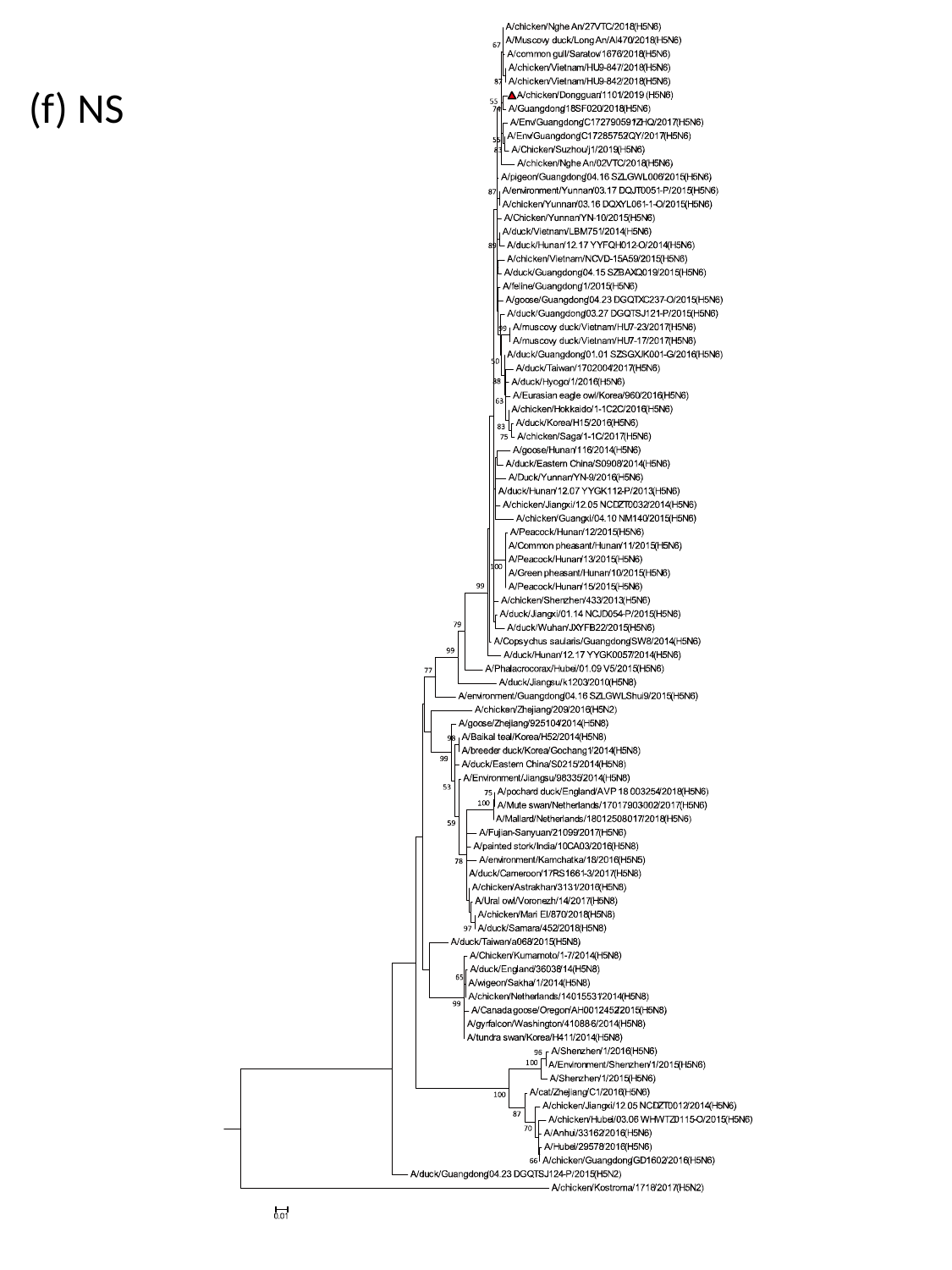

(f) NS
